# BRAFV600E mutation in high-grade dysplastic nevi: contributions to the distinction from early melanomas^؆^

**DOI:** 10.1016/j.abd.2026.501395

**Published:** 2026-06-16

**Authors:** Carolina Viza Amorim, Neilton Firmo de Lima, Camila Santos Saraiva de Moura Firmo, Leonardo Ávila Ferreira, Tatiane Iris Mancini, Israel Bendit, Maria Letícia Cintra, Luciana Nardinelli

**Affiliations:** a Department of Pathology, Medical Sciences School, Universidade Estadual de Campinas, Campinas, SP, Brazil; b Department of Dermatology, Medical Sciences School, Universidade Estadual de Campinas, Campinas, SP, Brazil; c Department of Hematology, Laboratory of Tumor Biology, Faculty of Medicine, Hospital das Clínicas, Universidade de São Paulo, São Paulo, SP, Brazil

Dear Editor,

Individuals with multiple dysplastic nevi (DN) are at increased risk for the development of cutaneous melanoma (CM).^1^ Their follow-up is essential. Also, it is possible that DN falls within an intermediate stage in CM progression.^2^ The distinction between DN and CM may be challenging, particularly in the presence of pronounced cytoarchitectural atypia, the so-called high-grade dysplastic nevi (HDN).^1^ Indeed, some excised lesions diagnosed as HDN could actually correspond to early-stage melanomas. Molecular markers have been investigated as auxiliary tools in distinguishing between benign and malignant lesions.^3^, ^4^ The BRAFV600E mutation promotes continuous stimulation of cell proliferation and survival. It is a common early event in melanocytogenesis, identified in up to 80% of acquired melanocytic nevi and in about 50% of CMs, particularly those in the vertical growth phase. However, the BRAFV600E mutation alone does not determine malignancy, as it induces a state of oncogenic senescence characterized by cell cycle arrest and blockage of tumor progression. For malignant transformation to occur, additional genetic events are required.^3^, ^5^

The BRAFV600E mutation is found in only 6% of intraepidermal CMs.^5^ Thus, detecting the mutation in lesions with marked atypia, but without dermal invasion, would favor HDN and could be an auxiliary tool in the differential diagnosis between HDN and incipient CM. We studied the rate of BRAFV600E gene mutations in a sample of lesions with a histopathological diagnosis of HDN, excised from individuals with dysplastic nevus syndrome.

From the archives of the Pathology Laboratory, all histopathological specimens diagnosed as DN, between 1994 and 2022, were retrieved. Clinical data from patients with DN syndrome were compiled. Two pathologists reviewed the histological sections. The following exclusion criteria were applied: 1) Samples from patients who had fewer than 3 nevi removed at the service; 2) Samples in which histopathological review raised doubts about differential diagnosis between HDN and CM; 3) Patients diagnosed with any genodermatosis. The selected specimens were those which presented: 1) Epidermal hyperplasia with a degree of irregularity and asymmetry; 2) Larger clusters of melanocytes at the dermoepidermal junction or more lentiginous areas; 3) Irregularities in the size, spacing, and polarity of melanocyte clusters and greater asymmetry in their distribution; 4) Uniform, fine (“dusty”) melanin pigment in the cytoplasm in some areas; 5) More pronounced and continuous cytological atypia, and 6) Asymmetry regarding hypervascularization, lymphoid infiltrate, and melanophages beneath the epidermis (Fig. 1).

**Fig. 1:**
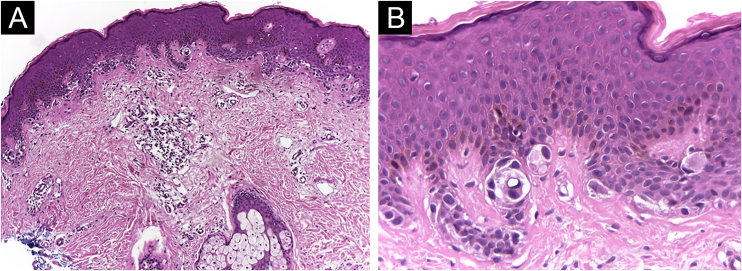
Junctional dysplastic melanocytic nevus with marked cytoarchitectural disorder. Hematoxylin & eosin, ×40 (A) and ×400 (B).

After applying the exclusion criteria, 25 HDN (9 patients, 5 male, 4 female) were included. Five 10 µm-thick lesions’ sections were cut with sterile blades and stored in 1.5 mL polypropylene tubes. DNA extraction was performed using the QIAamp DNA FFPE Tissue Kit (Qiagen, Germany), according to the manufacturer's protocol. The BRAFV600E mutation was screened by digital PCR using the QuantStudio 3D platform (Applied Biosystems, USA) and the TaqMan™ Liquid Biopsy dPCR Assay Hs000000004_rm; BRAF_476 (Applied Biosystems, USA).

Of the 25 HDN, 17 (68%) carried the BRAF mutation and 8 (32%) did not. All patients were white, and the mean age at diagnosis of the first DN was 27.8 years. Tables 1 and 2 show clinical data of patients and the lesions’ topography. All patients had at least one HDN with the BRAFV600E mutation, and 5 patients also had HDN without the mutation.

**Table 1:** Clinical data of the patients and number of HDNs.

Patient	Sex	Age^a^	Personal history of melanoma	Family history of melanoma	Personal and family history of melanoma	Total number of HGDNs	HDNs with mutation	HDNs without mutation
1	M	22	Yes	Yes (sister)	Yes	10	7	3
2	M	15	Yes	No	No	3	2	1
3	M	27	No	No	No	2	1	1
4	F	22	Yes	Yes (mother)	Yes	1	1	0
5	F	24	Yes	No	No	3	1	2
6	M	27	Yes	Yes (uncle)	Yes	1	1	0
7	F	45	Yes	No	No	1	1	0
8	F	24	Yes	Yes	Yes	1	1	0
9	M	45	Yes	No	No	3	2	1

a Age at diagnosis of the first dysplastic nevus.

**Table 2:** Dysplastic nevi with more pronounced cytoarchitectural atypia (*n* = 25): lesion topography.

Axial lesions	15 (60%)
Limb lesions	9 (36 %)
Lesion with unspecified location	1 (4 %)

Our results reflect the described higher prevalence of DNs in young, fair-skinned individuals, developing in photo-exposed areas, especially the trunk.^4^ The BRAFV600E mutation rate was similar to that described for DN in general (70%–80%), and much higher than that in early-stage melanomas (5%–10%).^3^, ^5^

Interestingly, only one of the nine patients had no personal history of melanoma. There is an association between multiple DNs and higher melanoma risk, although the chance of transformation for an isolated DN is low. This risk can be over 1200 times higher when there is a personal and family history of melanoma.^2^

Studies suggest that the absence of this mutation may be associated with more aggressive genetic alterations, such as NRAS and TERT mutations and CDKN2A deletions, indicating a molecular profile closer to incipient melanoma.^6^ This hypothesis highlights the importance of considering BRAFV600E-negative HDN as potentially higher-risk lesions.

Saroufim et al. reported a high prevalence of somatic BRAF mutations in dysplastic nevi, supporting the concept that BRAF activation represents an early event in melanocytic tumorigenesis. In addition, the observation of discordant BRAF mutational status among multiple dysplastic nevi within the same individual suggests that these mutations are independent somatic events rather than reflecting a constitutional predisposition, likely influenced by local environmental factors.^7^

There is still controversy regarding the follow-up of patients with a diagnosis of DN, because of the difficulty in distinguishing HDN from intraepidermal melanoma.^8^ In a multicenter study with 438 individuals, patients with two or more DN presented a significantly higher risk of developing melanoma in areas different from the DN biopsy. Therefore, regular full-body skin screening is important.^9^

This study has some limitations that should be acknowledged. The size of the study, determined by the number of included patients and analyzed dysplastic nevi, limits the generalizability of the findings. In addition, the absence of melanoma samples precludes direct diagnostic comparisons. Laser capture microdissection was not performed.

Overall, the integration of molecular, histopathological, and clinical data is essential for improving risk stratification and the management of high-grade dysplastic nevi.

## Authors' contributions

Carolina Viza Amorim: Writing of the manuscript, data collection, final approval of the final version of the manuscript.

Neilton Firmo de Lima: Data collection; final approval of the final version of the manuscript.

Camila Santos Saraiva de Moura Firmo: Data collection; final approval of the final version of the manuscript.

Leonardo Ávila Ferreira: Data collection; final approval of the final version of the manuscript.

Tatiane Iris Mancini: Data collection; final approval of the final version of the manuscript.

Israel Bendit: Data collection; final approval of the final version of the manuscript.

Maria Leticia Cintra: Writing of the manuscript, effective participation in the research guidance, final approval of the final version of the manuscript.

Luciana Nardinelli: Writing of the manuscript, data collection, manuscript critical review; final approval of the final version of the manuscript.

All authors have approved the final article.

## Financial support

None declared.

## Research data availability

The entire dataset supporting the results of this study was published in this article.

## Conflicts of interest

None declared.
